# Measurement Platform to Probe the Mechanism of Chiral-Induced Spin Selectivity through Direction-Dependent Magnetic Conductive Atomic Force Microscopy

**DOI:** 10.1021/acsnano.5c04980

**Published:** 2025-04-29

**Authors:** Joseph A. Albro, Noah T. Garrett, Keerthana Govindaraj, Brian P. Bloom, Nathaniel L. Rosi, David H. Waldeck

**Affiliations:** Department of Chemistry, University of Pittsburgh, 219 Parkman Avenue, Pittsburgh, Pennsylvania 15260, United States

**Keywords:** chiral-induced spin selectivity, chiral polyaniline fibers, magnetoresistance, spin polarization, direction dependent CISS

## Abstract

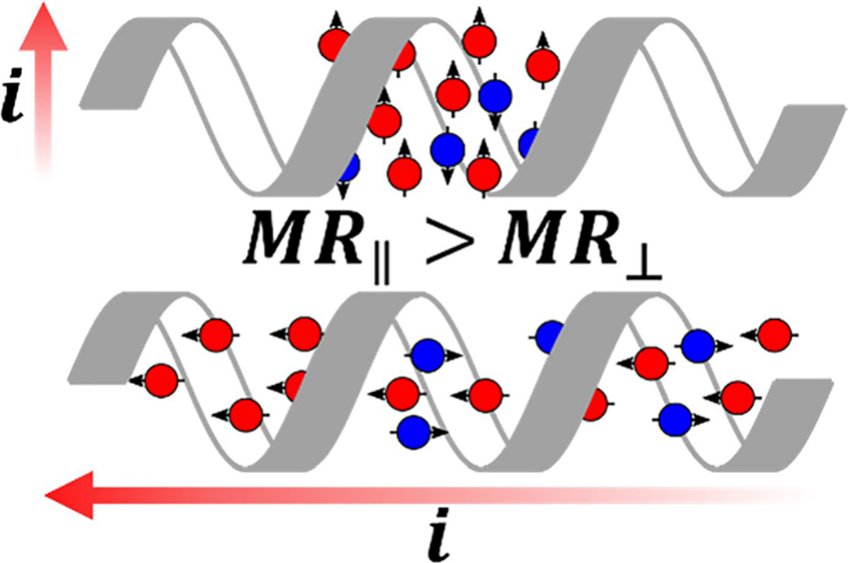

This work introduces a magnetic conductive atomic force microscopy (mc-AFM) measurement platform for determining spin polarizations, arising from the chiral-induced spin selectivity (CISS) effect along different directions in helical conducting fibers. By using the principle that the spin preference for electron transport in a chiral material changes with the momentum of the electron, this method quantifies the spin polarization of chiral materials, which straddle a ferromagnetic electrode, i.e., by taking measurements in regions to the right and left of the electrode while it is magnetized in-plane. The working mechanism of the measurement is shown using chiral polyaniline (PANI) fibers, and they reveal that the longitudinal, along the fiber’s helical axis, and transverse, perpendicular to the fiber axis, magnetoresistance differ by about a factor of 2. The observations imply that the spin polarization in PANI fibers is not consistent with models that attribute the spin selectivity (or magnetoresistance) solely to the spinterface or to spin-dependent charge injection barriers. In aggregate, this new platform offers a simplified approach for extending the mc-AFM method to resolving the spin-filtered charge currents along different directions in oriented samples.

## Introduction

The chiral-induced spin selectivity (CISS) effect refers to the inherent property of chiral materials to transport electrons with one spin orientation more favorably than that of the other spin orientation.^1^ The CISS effect was first reported for the transmission of spin-polarized photoelectrons through Langmuir–Blodgett films of stearoyl-lysine^2^ and has since been reported for a wide array of chiral molecules, biomolecules, and inorganic chiral materials.^3^ Moreover, the spin selectivity also manifests for electron transport below the vacuum level (i.e., tunneling, hopping conduction, etc.) and for the charge polarization of chiral molecules and chiral materials. CISS is providing new insight into biological processes,^4−9^ new approaches for controlling electrochemical reactions and interactions,^10−17^ and new design elements for spin-(opto)electronics,^17−21^ among others.

While considerable efforts have been dedicated to developing a theoretical understanding of CISS, the underlying mechanism is still debated^22^ and experiments that probe new aspects of the phenomenon are needed. Early models for CISS in molecules attributed the phenomenon to the electron’s spin–orbit coupling (SOC), however those early models failed to reproduce the large spin selectivity that was measured experimentally, unless the SOC was made unusually large compared to that inferred from orbital models.^22^ More recent approaches, which include many body effects, electron–electron correlation, electron–phonon coupling, and models more akin to Rashba or Rashba–Edelstein ideas provide more realistic descriptions.^3,23−25^ Given that most measurements of CISS have been with electrode-chiral molecule (or material) interfaces, other models have focused on the interface and proposed that magnetochiral interactions with the substrate affect the charge current measurement.^26−29^ For example, the ‘spinterface’ model invokes the substrate’s SOC, which can be significantly larger than that of chiral organic molecules, to generate large spin polarizations. In a similar vein, a recent model proposes that the experimentally observed changes in current–voltage (*i–V*) characteristics with magnetic field direction arise from changes in the barrier height for spin injection at the ferromagnetic electrode-chiral molecule interface.^30^ In this work, we design a platform for magnetic conductive-atomic force microscopy (mc-AFM) measurements that explores the importance of the interface (or spinterface) on the *i–V* characteristics in a new way.

First introduced in 2011,^31^ and now one of the more common methods used for measuring the spin selectivity of chiral films, is magnetic conductive atomic force microscopy.^13^ In this method, either the substrate or the AFM tip is ferromagnetic and used as an ‘analyzer’ to measure the change in *i–V* characteristics when magnetized with the North or South pole of a magnet. These measurements have been performed in a vertical geometry, along the normal to the substrate surface, even though the molecule or material’s helical symmetry axis may not be oriented out-of-plane, i.e., along the transport path for the measured current. Although many works show that CISS in helical chiral molecules is maximized along the helical axis,^32−34^ this is not always true,^35^ and measurements of the orientation dependence of the spin-polarized electron current are rare. To accommodate geometries with a longitudinal helical axis for a material some researchers have constructed two-terminal devices,^36,37^ a process which can require demanding and time-consuming fabrication techniques. Herein, we report a platform for measuring the spin dependence of both the transverse and the longitudinal charge current through helical fibers of polyaniline (PANI), using the mc-AFM method; see Figure S1 for an illustration of the molecular structure of chiral PANI. In applying this method to the spin-dependent electron transport through chiral PANI fibers, we find that the spin polarization of the electron current along the fiber’s helical axis is about twice that of the spin polarization perpendicular to its chiral axis, thus demonstrating that the spin filtering depends on direction in a chiral material.

### Measurement Design

**Figure 1:**
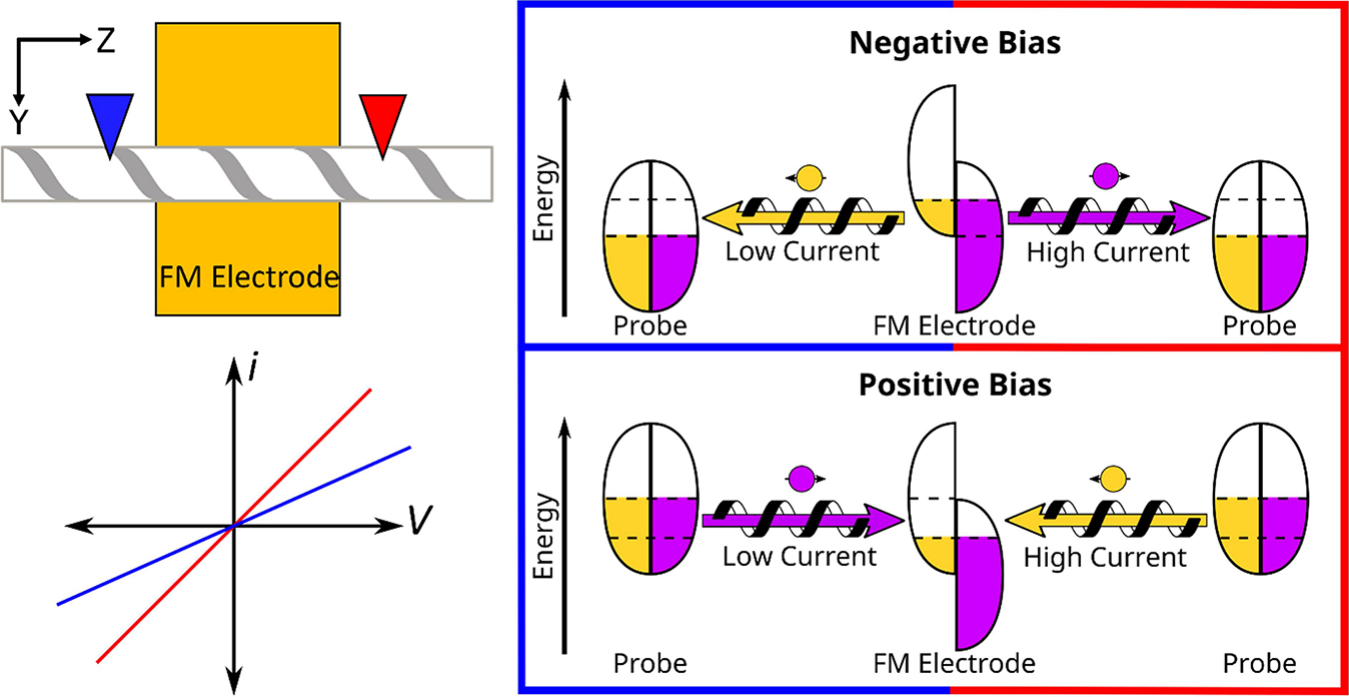
Scheme that illustrates the principles for longitudinal spin polarization measurements using mc-AFM with the magnetic field oriented along the +*Z* direction. The blue and red triangles, which represent AFM tip locations, and corresponding red and blue outlines, represent separate measurements in different regions of a chiral fiber (the white rectangle with a gray ribbon), to the left (blue) and right (red) of a ferromagnetic (FM) electrode. The electron spin orientations in the lab frame are distinguished by ‘pink’ (oriented along the field direction) and ‘yellow’ (oriented against the field direction) colors in the diagram. The spin preference of the chiral material depends on the momentum of the electron. For the case shown, it is assumed that the chiral fiber selects for spins aligned parallel to the electron momentum direction. The momentum direction of the electron is denoted by the colored arrow inside of the helix, and the color of the arrow represents the spin preference of the electron in the lab frame. The sketch of the expected current–voltage (*i–V*) curves in the bottom left illustrates the expected *i–V* values at the blue and red AFM tips in the ohmic limit.

**Figure 2:**
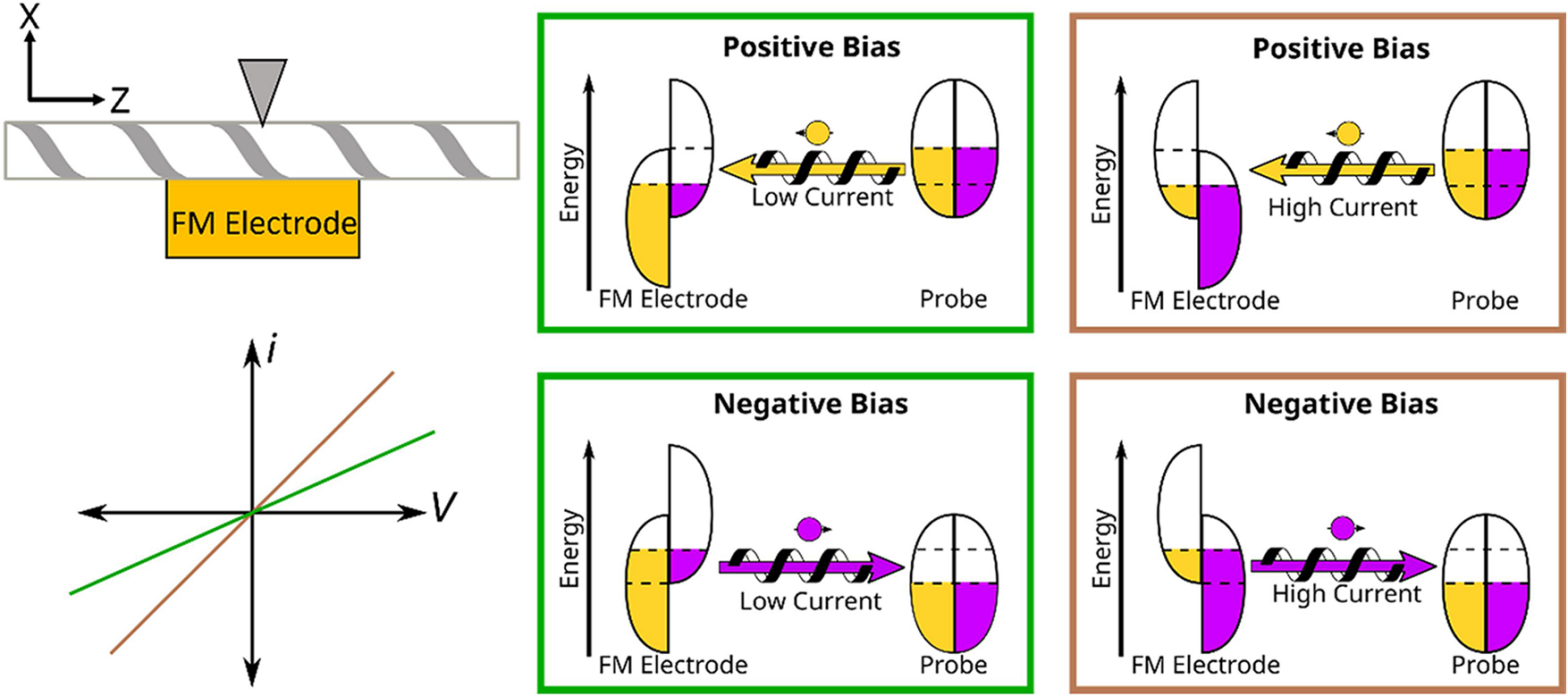
Scheme that illustrates the principles for transverse spin polarization measurements using mc-AFM. Note that the transverse measurements are performed by placing the conducting AFM tip directly above the ferromagnetic electrode. The current–voltage diagram shows how the *i–V* curves are expected to differ for the two different magnetic field orientations along the *X*-axis (brown (B(+*X*)) and green (B(−*X*))). The insets in the boxes illustrate the underlying cause for the change in current with the different voltage bias and magnetization conditions. As with the longitudinal diagram, the electron spins are distinguished by the ‘pink’ and ‘yellow’ colors for the different experimental parameters. Spin-dependent currents are additionally shown as arrows to and from the spin sub-bands.

Measurements of the CISS effect imply that the spin selectivity of a chiral material is determined by the electron spin direction and the electron’s linear momentum. This work leverages the change in spin preference with the momentum of the electron to measure the spin polarization of a chiral material whose helical axis lies in-plane, referred to as a longitudinal measurement geometry and shown in Figure 1. In addition, the measurement platform can accommodate spin-polarized charge current along the direction perpendicular to a material’s helical axis, referred to as ‘transverse’ and shown in Figure 2.^38,39^

To understand the operating principle of the longitudinal measurement, consider a chiral material, represented by a helix, that straddles a ferromagnetic (FM) electrode; see Figure 1. Because the linear momentum of the electron is coupled to the preferred spin for transport in a chiral material, a change in the sign of the momentum of the electron should cause a change in spin preference. Therefore, mc-AFM measurements made on a chiral fiber to the left (blue) and right (red) of an FM electrode, which is magnetized along *Z*, will probe an opposite spin direction, in the lab frame. In this way, the spin polarization of a material can be determined without changing the FM’s magnetization direction. Figure 1 shows the case for magnetization of the FM along B(+*Z*) with a chiral material that preferentially transmits electrons with spins oriented parallel to their momentum. Upon magnetization, the spin sub-bands in the FM split and the resulting imbalance in spin population is indicated by the difference in the areas of the yellow and pink regions. When the FM electrode is biased positive, relative to the nonmagnetic AFM tip, the Fermi level decreases and electrons flow from the tip to the FM electrode. Performing the *i–V* measurement to the left (blue) selects for the pink spins, because the chiral fiber selects for parallel spin orientation; and measurements to the right (red) select for yellow spins, because the chiral fiber selects for the parallel spin orientation. Because the pink sub-band is the majority spin, the blue AFM tip location leads to more resistive transport (lower current) than the red AFM tip location which preferentially probes ‘yellow’ spins. The bottom left diagram indicates this difference by a higher current for the red trace than the blue trace under positive bias. When the bias is reversed, i.e. negative bias, electrons flow from the FM electrode to the AFM tip. Because the electron momentum direction changes, the spin preference (yellow versus pink) flips, but is still parallel to the momentum in the chiral material. Because the AFM tip on the right (red) samples the majority spins in the FM electrode, it is less resistive, i.e., shows a higher current.

In contrast to the traditional method of changing the magnetization direction of the electrode to probe the spin-dependence of the electron transport, the method described above uses a single magnetization direction for the electrode, but samples the current at different ends of the fiber. This approach allows one to rapidly make multiple measurements of the spin-dependent current with a fixed geometry. By performing the measurements with the magnetic field oriented along +*Z* and −*Z*, however, it is possible to extract the spin-dependent anisotropy in the electron current in a manner that is directly comparable to that used in the traditional measurement along the normal to the FM electrode, see Figure 2. Comparison of data for these two measurement types is discussed in the results section, *vide infra*.

Now consider a geometry for a traditional transverse measurement, in which the electrode is magnetized in the B(X) direction and the AFM tip is placed above the fiber atop the FM electrode (see Figure 2). Magnetization of the FM along B(+*X*) once again splits the spin sub-bands, however the majority spins are now oriented along the surface normal, along X (brown). When the FM is biased positive relative to the tip, electrons will flow from the tip, through the fiber, and into the FM. With a parallel spin-momentum transport preference, the chiral material will select for yellow spins. Because the FM is magnetized with a pink spin majority, it preferentially accepts the yellow spins, resulting in a high current. Inverting the magnetization direction (green) will change the spin imbalance in the FM; however, the spin preference of the fiber is the same, preferring yellow spins. Because the yellow spins are now the majority spins in the FM, a low current results. This difference is represented in the sketch by showing a higher current for the brown curve than the green curve under positive bias. Next, consider the negative bias case, which reverses the electron momentum direction. When the FM is magnetized with a yellow spin majority, it will provide more yellow spins than pink spins. Because the electron momentum direction is reversed, however, the fiber now preferentially transports pink electrons and a low current results. If the magnetization is changed to create a pink spin majority at the FM, a higher current results since it matches the spin preference of the fiber. The difference in response with magnetization is reflected in the *i–V* curves; a larger negative current for the brown case than for the green case. In this way, the spin-dependent electron current in the transverse direction can be measured by changing the magnetization of the FM.

The spin-dependent electron currents that are measured with the longitudinal geometry, to the left and right of the FM electrode, are akin to that of the conventional out-of-plane mc-AFM measurements performed with +*X* and −*X* applied magnetic fields. Here we note some differences in the underlying assumptions and implementation for the longitudinal and transverse measurements. A spin-dependent anisotropy in the current can be quantified in longitudinal measurements so long as (i) the conductivity of the chiral fiber is the same on both sides of the electrode and (ii) the magnitude of the spin preference is equivalent on both sides. Note that similar constraints occur in transverse measurements, e.g., location dependent changes in the conductivity of a material can distort the calculation of spin polarization. For this reason, transverse measurements were performed in similar locations for both +*X* and −*X* magnetizations and large sampling sizes, >30 measurement locations were sampled and averaged.

## Results and Discussion

**Figure 3:**
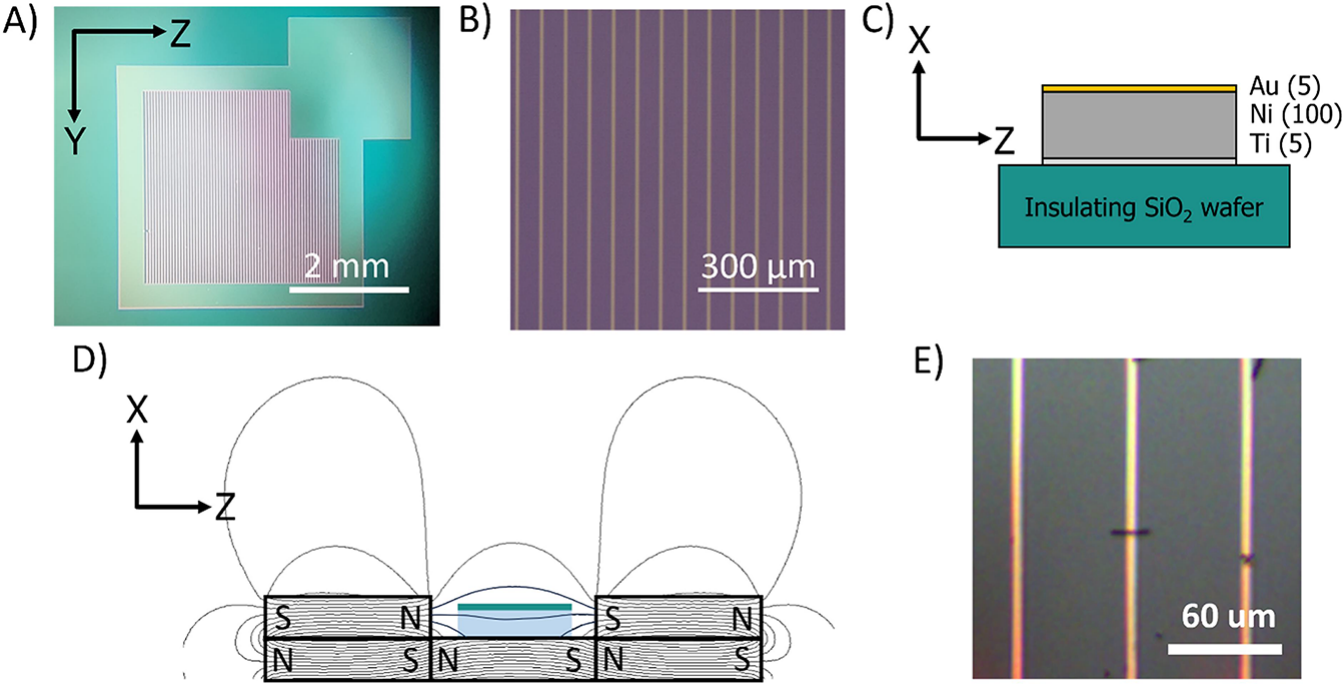
Measurement setup and implementation. (A and B) Top-down optical images of the FM electrode device platform. The *Z*-direction in the laboratory frame is perpendicular to the electrode trace, and the *Y*-direction is along the FM electrode’s long axis. (C) Side-view for one of the FM electrode traces and defines the *X*-direction in the lab frame, along the sample’s surface normal. (D) Magnetic field diagram for five magnets on the AFM sample chuck, which holds the sample, and the calculated magnetic field lines. An in-plane magnetic field acts across the device. (E) Optical image of a drop-casted fiber that is oriented perpendicular to the electrode, along *Z*.

The measurement platform for longitudinal and transverse mc-AFM was fabricated using photolithography on a silicon wafer with a 500 nm thick thermal oxide. The ferromagnetic (FM) electrodes comprise a 5 nm Ti adhesion layer, 100 nm Ni layer, and a 5 nm Au layer to inhibit oxidation of the underlying Ni; see Methods section for additional details. The device possesses a series of 4 mm long and 5 μm wide FM strips that are spaced by 55 μm and connected to a common bus for electrical contact. Figure 3A,B shows a top-down optical microscope image of the device, and Figure 3C shows a side view illustration. During measurement a static magnetic field was applied in-plane along the *Z*-axis of the device using a stage comprising a series of permanent magnets for longitudinal measurements, or by placement of a permanent magnet directly under the electrode for transverse measurements along the *X*-axis. Figure 3D shows a magnetic circuit diagram of the sample stage for the longitudinal measurements, with a mounted measurement platform, generated using the Finite Element Method Magnetics program.^40^ Note that a magnetic field oriented along *Z* is applied across the device in this configuration; see Figure S2 for a vector plot of the magnetic field. In the longitudinal geometry the magnetic field at the sample position was measured to be 136 mT and in the transverse geometry the magnetic field was measured to be 360 mT near the sample plane. These fields are large enough that they are many times the coercive field of Ni films, ∼10 mT.^41^

To determine the efficacy of the measurement system, chiral polyaniline (PANI) fibers, previously reported to exhibit longitudinal spin polarizations through 2-terminal device measurements,^36^ were chosen as a model system. Figure S1 shows the absorbance and circular dichroism of R- and S-PANI fibers, and Figure S3 shows corresponding SEM images which highlight the secondary structure that manifests in the materials; see Methods and Supporting Information for additional information. The PANI fibers were drop-cast onto the surface of the device and the optical microscope of the AFM was used to locate a fiber that was lying in-plane and oriented perpendicular to the FM electrode; see Figure 3E. Additionally, only fibers whose morphology did not exhibit twisting of the secondary structure in the region directly above the electrode were considered in order to ensure optimal contact. The in-plane/longitudinal mc-AFM of a single chiral PANI fiber was acquired through the following measurement sequence:

(i) The AFM tip was placed on a region of the fiber to the left of the FM electrode and five *i–V* curves were collected.
(ii) The AFM tip was placed on a region of the fiber to the right of the FM electrode, approximately equidistant from the electrode as in (i), and five *i–V* curves were collected.
(iii) Processes (i) and (ii) were repeated for a minimum of three times at a given distance from the FM electrode.
(iv) Steps (i)–(iii) were repeated for at least a total of four times, with the tip placed at a new set of locations each time.

The purpose of this measurement sequence is 2-fold. First, multiple measurements at different points in a given region of the chiral material mitigate the impact of material conductivity changes that might cause systematic error in the calculated spin polarization. This is analogous to what has been done for out-of-plane/transverse mc-AFM measurements. Second, making sequential measurements to the left and right of the FM electrode minimizes the effect of tip wear or fouling on the determined spin polarization. Progressive tip wear or fouling of the AFM tip can lead to progressive changes in the measured *i–V* characteristics with measurement time or even a null response in current. Through our sampling method the effects of tip wear are treated equally for measurements made on the left and right of the FM; and in the event of contamination all data prior to its occurrence can still be considered. The protocol for out-of-plane/transverse geometry is to collect *i–V* data for the magnetic field along +*X*, then *–X*, and finally +*X*. In this scheme the data are considered to be valid if the first and second +*X* measurements agree, however, there is no way to determine the effects of tip wear or contamination until the measurement sequence is completed.

**Figure 4:**
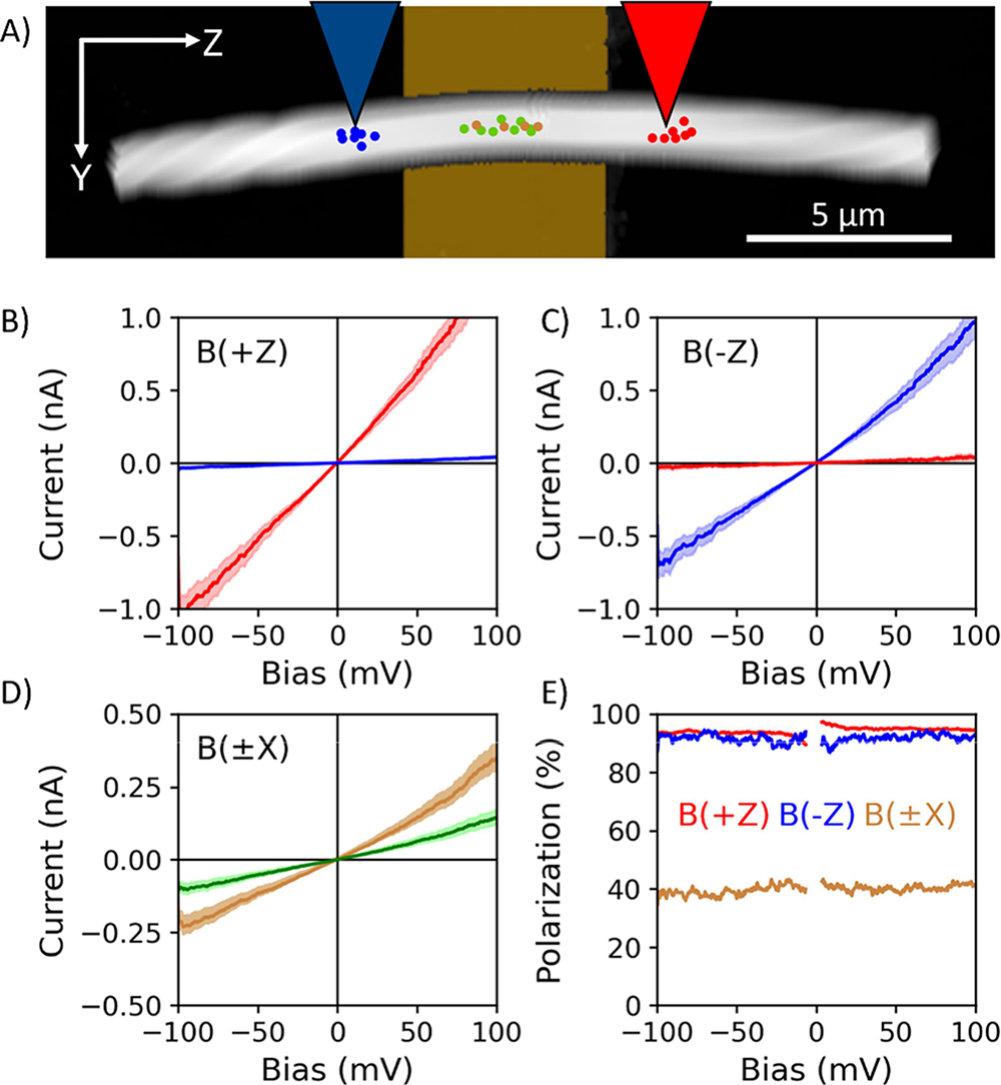
Measurement of an S-PANI fiber. (A) AFM topography of the measured S-PANI fiber. Locations of longitudinal *i–V* measurements are shown by red and blue dots. The locations for the transverse *i–V* measurements are shown by green and brown dots and are approximately in the middle of the FM electrode. Blue and red colored AFM tips depict the side of the electrode corresponding to results shown in panels (B) and (C). (B) Average of 120 *i–V* curves from 4 different locations for each side of the fiber for the magnetic field oriented in the +*Z* direction, B(+*Z*). (C) Average of 120 *i–V* curves for the *–Z* direction, B(*−Z*). (D) Average of >120 *i–V* curves with magnetization out-of-plane along the *X* direction; B(+*X*) and B(*−X*) are represented by brown and green curves, respectively. The shaded region in all of the curves represents 95% confidence intervals to the average data. (E) Spin polarization as a function of sample bias.

Figure 4 shows *i–V* data that were collected using the in-plane geometry with both B(+*Z*) and B(*−Z*) magnetic fields and using the out-of-plane geometry with B(+*X*) and B(*−X*) magnetic field directions, on an S-PANI fiber. Figure 4A shows an AFM topography image of the fiber and the location where the *i–V* curves are collected is indicated by the color: red, to the right of the FM electrode, or blue, to the left of the FM electrode for longitudinal measurements and green and brown for B(*−X*) and B(+*X*) for transverse measurements. Panel B shows the average of 120 *i–V* curves (solid line) for the two different regions collected with a B(+*Z*) magnetic field. The shaded region corresponds to the 95% confidence interval of the data. A higher current is observed when the momentum of the electron is parallel to its spin (red) compared to when the momentum of the electron is antiparallel to its spin (blue) and reflects the CISS response of the fiber. Note that 4 times out of 120, the current on the left (blue) was below the 5 pA current detection threshold and could not be recorded, but for measurements on the right (red) side all 120 measurements gave measurable currents. Experiments were then repeated on the same fiber with a magnetic field applied along B(*−Z*); see Figure 4C. Here, the change in magnetization across the device changes the ferromagnetic analyzer’s spin preference such that the blue and red curves now correspond to parallel and antiparallel, respectively. A higher current is again observed when the electron spin is parallel to its transport direction. In this case, all 120 measurements on the left (blue) side yielded a measurable signal, but 92 times out of 120, the current on the right (red) was below the 5 pA detection threshold of the instrument and was therefore not recorded. We attribute the sub 5 pA current response to a very high spin-filtering. If the measurements for B(+*Z*) are scaled down according to the difference in average current value between B(+*Z*) and B(*−Z*), 55 of 120 measurements would not show an average value greater than 5 pA, which could indicate that tip degradation may have pushed more of the measurements for B(*−Z*) below the 5 pA measurement threshold.

The anisotropy in the longitudinal charge current *i*, which we call spin polarization *P*, was quantified by
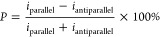
1In the case of a magnetic field applied along the +*Z*-direction, B(+*Z*), the majority electron spin state of the electrode has spins oriented along the applied field direction. If a chiral fiber selects for spins parallel to their momentum, then it will display a larger current on the right than on the left. The spin polarization with B(+*Z*) was found to be 94 ± 0.8% and largely independent of the bias voltage; see the red curve in Figure 4E. For a magnetic field oriented along *–Z*, B(*−Z*), the opposite current response in the lab frame is expected if the chiral molecule selects for the parallel alignment of the electron spin with the electron momentum, see Figure 4C. The spin polarization with B(*−Z*) was found to be 92 ± 3.3% in this case. Thus, the spin polarization with B(+*Z*) and B(*−Z*) were found to be approximately equal, as expected for the measurement mechanism described above. If CISS is responsible for the spin selection then we expect that the opposite spin preference should be observed for R-PANI fibers. This prediction was validated in a separate set of experiments and the data are presented in Figure S4.

The above analysis uses the charge currents along the two different directions of the fiber to calculate the spin polarization; however, it is also possible to calculate the anisotropy that results from the change in current with electrode magnetization, which we call magnetoresistance, MR. The percent MR is defined as
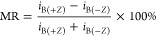
2where *i*_B(+*Z*)_ and *i*_B(−*Z*)_ are the currents measured at the same position along the fiber under the two different magnetic field directions. Using the measured data in Figure 4B,C for the measurements on the right (red) side of the fiber, we obtain an MR of 94 ± 1.6%; and if we use the currents measured on the left (blue) side of the fiber, we obtain an MR of −91 ± 1.2%. The values for MR(red) and MR(blue) are found to be approximately equal in magnitude, while the sign of the MR is opposite between sides of the fiber. This is expected, as the fiber only selects for one spin-momentum alignment, and opposite sides of the fiber are measuring opposite spin-momentum alignments. The MR magnitudes show that the measurement itself is consistent with that expected from traditional mc-AFM measurements in which the magnetic field dependence is used to extract a value; however, the method used in Figure 4B,C only requires a single magnetic field direction to be applied in order to quantify a spin polarization.

Now consider a geometry for transverse (out-of-plane) measurements on the same fiber in Figure 4. Here, the magnetic field was oriented along the *X*-direction, and the spin polarization was quantified from *i–V* curves of B(+*X*), brown trace in Figure 4D, and B(*−X*), green trace in Figure 4D. Figure 4E shows a plot for the polarization/MR of the PANI fiber measured in the transverse geometry (brown curve). The same sign of polarization is observed with the two measurement geometries, however measurements perpendicular to the helical axis of the fiber exhibit a different polarization magnitude (40 ± 4.9%). Note that the sign of the spin polarization agrees with that shown in out-of-plane measurements on similar PANI fibers.^42^ Because previous studies have shown that increasing transport length through chiral materials can lead to higher spin polarizations, longitudinal measurements were performed at distances of ∼1 μm away from the FM electrode, approximately the same as the thickness of the PANI fiber. We therefore attribute the difference in spin polarization to the orientation of transport with respect to the chiral helical axis of the fiber and not the transport length. Note, Figure S5 shows a series of measurements like that described for Figure 4 on a different fiber that corroborate these findings, albeit with different magnitudes for the polarization.

How does the measurement platform probe the mechanism of CISS? The fact that the direction of current flow through the long axis of the chiral fiber correlates with the MR measured on either side of the fiber supports a CISS mechanism in which the transport through the chiral material affects the spin selectivity, rather than a pure interface effect. In these experiments, the fiber is drop cast onto the FM electrode and the interface is dominated by the 5 μm by 1 μm area on the top of the electrode, compared to the side walls whose area is 50 times smaller and should have less physical contact. Thus, the interface is the same for both the B(+*Z*) and B(*−Z*) measurements with the only difference being spin alignment in the plane of the FM electrode, not perpendicular to its surface. Note that these studies are performed at distances well beyond the tunneling regime and that these conclusions may be different for other transport mechanisms, such as tunneling. Further research on the impact of the transport mechanism on the CISS response is beyond the scope of the current study.

**Figure 5:**
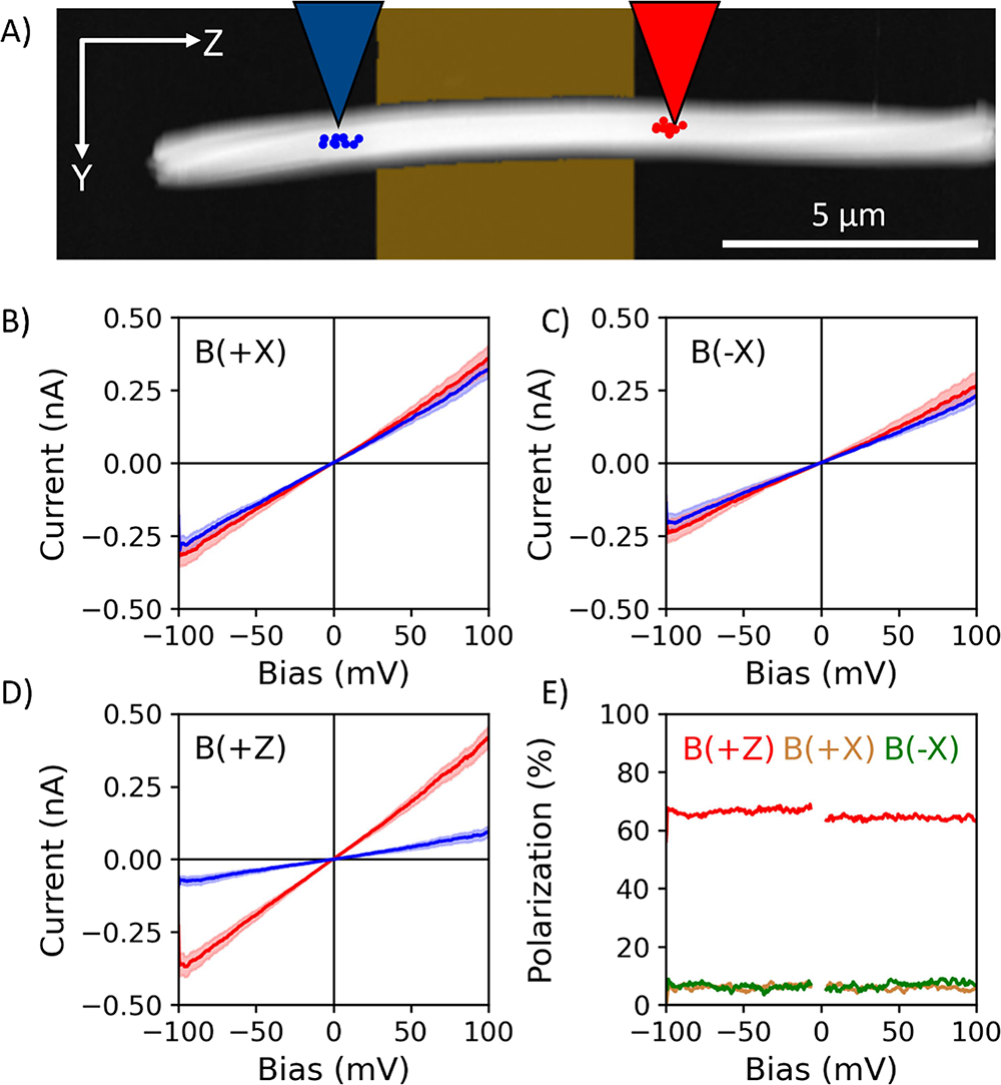
Longitudinal measurements of an S-PANI fiber under multiple field directions. (A) AFM topography of the measured S-PANI fiber. Locations of longitudinal *i–V* measurements are shown by red and blue dots. Blue and red colored AFM tips depict the side of the electrode corresponding to results shown in panels (B), (C), and (D). An average of 120 *i–V* curves from 4 different locations for each side of the fiber is reported for the magnetic field oriented in the (B) +*X* direction, B(+*X*) and (C) *–X* direction, B(*−X*). (D) Average of 120 *i–V* curves from 4 different locations for each side of the fiber with the magnetic field oriented in the +*Z* direction. The shaded region in all of the curves represents 95% confidence intervals to the average data. (E) Graph of the spin polarization plotted as a function of sample bias.

The dependence of the spin-momentum alignment was further tested by measuring the current through a fiber at locations approximately 1 μm on either side of the electrode under external magnetic fields of B(+*Z*), B(+*X*), and B(*−X*) (Figure 5). The case for B(+*Z*) in Figure 5D is like that described in Figure 4B, and this fiber displays a polarization of 65 ± 4.2%. For B(+*X*) and B(*−X*), however, the spin of the injected electron is oriented perpendicular to the helical axis of the fiber but the current is sampled about 1 μm past the electrode along *Z*; i.e., along the fiber’s helical axis. Thus, there is no symmetry breaking from the ferromagnetic electrode along the chiral symmetry axis, i.e., the Z-components of the injected electron spin are equally likely to be +*Z* as *–Z*, and no difference in measured current on either side of the electrode is found. B(+*X*) and B(*−X*) exhibit spin polarizations of 6.0 ± 4.9 and 6.6 ± 5.4%, respectively, and demonstrate that the spin polarization is largely nonexistent under magnetic fields that do not contain a symmetry breaking component along the direction of electron transport. The values found for the magnetoresistance (16 ± 5.8 and 16 ± 4.2%) are attributed to tip degradation, as the values are similar in magnitude and sign of those obtained from a similar sequence of measurement on an R-PANI fiber (Figure S6).

## Conclusions

This work describes a measurement scheme for determining longitudinal CISS-mediated spin polarizations with mc-AFM. A measurement platform was constructed, and its working principle was demonstrated using chiral polyaniline fibers. The data show that the spin polarization of the PANI fiber is maximized along the helical axis of the fiber and that the response does not originate solely from spin-interface related phenomena. Collectively, this work offers a simplified approach for measuring spin-dependent charge transport along different directions in chiral materials and provides new insights into the working mechanism of CISS.

## Methods

### Synthesis of PANI Fibers

#### Reagents

*S*-camphor sulfonic acid (S-CSA) (Sigma-Aldrich 99%), *R*-camphor sulfonic acid (R-CSA) (Sigma-Aldrich 99%), 2,3-dichloro-5,6-dicyano-1,4-benzoquinone (DDQ) (Sigma-Aldrich 98%), *n*-phenyl–phenylenediamine (Alfa Aesar 98%), and methanol (MeOH) (Fisher Chemical) were used as received. Tetrahydrofuran (THF) (Fisher Chemical) and chloroform (CHCl_3_) (Fisher Chemical) were freshly distilled to remove stabilizers before use. Aniline (99+% Thermo Scientific) was distilled (2×) under reduced pressure immediately before use.

#### Polyaniline

*R*- or *S*-CSA (290.3 mg; 1.25 mmol), CHCl_3_ (4.9 mL), 0.1 mL of 0.08 M *n*-phenyl–phenylenediamine in CHCl_3_, and aniline (58 mg; 0.625 mmol) were added in sequence to a 20 mL scintillation vial. The vial was capped and shaken for ∼5 min to completely dissolve all contents. The solution was allowed to sit for 1 h at 25 °C. Then, 1.675 mL of 0.373 M DDQ in THF was added to initiate polymerization and the resulting solution was shaken for ∼5 min. The vial was left undisturbed for 16 h at 25 °C.

#### Fiber Formation

THF (1.4 mL), CHCl_3_ (4.2 mL), polyaniline (0.2 mL of above solution), and MeOH (10.4 mL) were added to a 20 mL scintillation vial. The vial was capped and shaken for ∼5 min. The solution was allowed to sit undisturbed for 24 h to yield PANI microfibers as a green precipitate. An aliquot of the as-synthesized precipitated PANI microfibers was collected for UV–Vis and circular dichroism (CD) measurements. The supernatant was carefully removed and replaced with fresh MeOH (3×) to wash the fibers prior to electron microscopy and AFM measurements.

### Characterization of PANI Fibers

#### Scanning Electron Microscopy (SEM)

SEM imaging was conducted on a Zeiss 500 VP scanning electron microscope. Samples were prepared in MeOH and drop-cast onto a silicon wafer. The samples were dried under ambient conditions and loose material was displaced by a stream of N_2_ gas before imaging. Images were collected at 1 kV and at a working distance between 3 and 6 mm.

#### UV–Vis and CD Spectroscopy

PANI fibers dispersed in a mixture of THF and CHCl_3_ within a 1 cm path length quartz cuvette. UV–Vis spectra were collected on an Agilent 8453 UV–Vis spectrometer that was equipped with deuterium and tungsten lamps. CD spectra were collected on a JASCO 810 instrument from 260 to 800 at 50 nm/min with two iterations.

### Fabrication of Longitudinal Measurement Platform

The electrodes were fabricated on a Si wafer with a 500 nm wet thermal oxide layer (University Wafer inc., item 1176) using photolithography. The photolithography used a bilayer photoresist technique, with the bottom layer consisting of Kayaku LOR 5B, spin coated at 4000 rpm and then heated to 195 C for 9 min, and a top layer, comprising Kayaku MICROPOSIT S1805 G2, spin coated at 4000 rpm and then heated to 115 °C for 3 min. The photolithography was performed with a Heidelberg MLA100 Direct Write system. The devices were developed in a MICROPOSIT 351 developer, diluted with deionized (DI) water 1:4, for 75 s, followed by rinsing with DI water, and then developed in AZ 400k 1:4 for 45 s, once again followed by rinsing in DI water and drying in nitrogen.

Following the development process, the device was placed into a Plassys Electron Beam Evaporator MEB550S, where it was subjected to Ar plasma cleaning at 250 V and 15 A for 60 s. The layers for the deposition comprised an adhesion layer, consisting of 5 nm of Ti deposited at 0.02 nm/s, a magnetic layer, consisting of 100 nm of Ni deposited at 0.2 nm/s, and a layer of 5 nm of Au deposited at 0.05 nm/s, to prevent oxidation of the underlying magnetic layer. After deposition, the wafer was placed overnight in Kayaku Remover PG to allow for liftoff of the remaining photoresist, leaving behind the metal deposited directly onto the wafer while removing the metal coating the photoresist.

The devices were then coated in a layer of MICROPOSIT S1827 photoresist and then diced by an Advanced Dicing Technology (ADT) 7122 dicing machine. Immediately following dicing, the electrodes were cleaned by twice sonicating in acetone for 10 min, changing the solution and rinsing with isopropyl alcohol in between sonications, and then sonicated for 15 min in Kayaku Remover PG. After sonication, the electrodes were rinsed with isopropyl alcohol, and then dried with nitrogen.

### mc-AFM Measurements

#### Sample Deposition

A suspension of PANI fibers in MeOH (20 μL, 0.1 mg/mL) was drop-cast onto the fabricated device, and the MeOH was allowed to evaporate. The device was then fixed to a vertical 45° mount and 10 μL (3×) of MeOH was aliquoted to the top of the device such that the solvent flowed perpendicular to the direction of the electrodes. Pooled solvent was removed with a micropipette and the device was stored in a vacuum desiccator before AFM measurement.

To generate a magnetic field in plane, the sample was placed in a magnet assembly consisting of five 1/8-in. × 1/2-in. × 1-in. magnets for which the poles emanate through the 1/2-in. dimension. The magnets were epoxied onto a stainless steel plate (see Figure 3D) which was mounted onto the sample chuck of the AFM using double sided Kapton tape. The magnets were purchased from KJ Magnetics Inc. (item BX028). The magnetic field was measured to be 136 mT at the center of the sample area of the magnetic assembly using a hand-held Gaussmeter.

AFM measurements were performed with a Bruker Icon SPM that is equipped with Peak Force TUNA using a MicroMasch HQ:NSC-18/Pt probe. The sample was placed in the magnetic assembly with double sided Kapton tape and electrical connection was made to the AFM chuck through a 29 AWG flexible wire (McMaster Carr, item 9564T2) approximately three inches in length, attached to the sample using silver paint (Ted Pella, Inc., PELCO conductive silver paint, item 16062). The opposite end of the wire was attached directly to the AFM chuck using copper tape to hold it in place. The connection was confirmed using a hand-held multimeter by measuring the continuity between the AFM chuck and the far edge of the electrical bus of the sample.

After an initial imaging scan, locations for *i–V* curves were chosen using the point and shoot software module at positions approximately equidistant from the electrode. The deflection set point was chosen such that approximately 15 nN of force was applied at a given location. The bias was varied from −0.1 to 0.1 V during the collection of *i–V* curves and five *i–V* curves were performed at each location. The AFM tip was placed sequentially on each side of the ferromagnetic electrode for four cycles; i.e., five *i–V* curves were collected on the left, then the right and this was repeated three times. This procedure gave 20 *i–V* curves on the left and 20 *i–V* curves on the right. The process was then repeated in a new set of locations. After all the *i–V* curves were collected, another imaging scan was taken to ensure that there was no sample drift that would have affected the measurement.

The *i–V* curves were first examined and curves that did not produce any current above the detection threshold of the instrument (∼±5 pA) were discarded. For measurements with currents below this threshold, the vast majority were from the “unpreferred” side of the fiber. Removing these curves from the results bias the calculated spin polarization to lower values. Curves were then sorted by location relative to the electrode. The average value of the two groups of curves was plotted and the 95% confidence interval was calculated. From the averaged values, we calculate the spin polarization as a function of the bias.

Uncertainty in the spin polarization was determined by , where σ_P_ is the error in the polarization, σ_*i*_(A)P__ is the standard deviation of the mean of the current at a particular bias on the side of the fiber measuring (anti)parallel spin momentum alignment, and  is the partial derivative of eq 1 with respect to the current measuring (anti)parallel spin momentum alignment.^43^ The uncertainty as a function of bias voltage was then averaged to give an average error for spin polarization.

Further note that while a single fiber exhibits a robust spin polarization, there is a significant fiber to fiber variability in spin polarization. As such, each set of measurements were performed on a singular fiber.

## Supporting Information Available

Chemical structure of chiral PANI, absorbance, circular dichroism, magnetic field vector simulation of platform stage, SEM of PANI fibers, and additional transverse and longitudinal measurements of PANI fibers (PDF)
