# Supplementary material for: Measurement Platform to Probe the Mechanism of Chiral-Induced Spin Selectivity through Direction-Dependent Magnetic Conductive Atomic Force Microscopy
Source: ACS Nano. 2025 Apr 29;19(18):17941–9. doi: 10.1021/acsnano.5c04980 (PMC12080373; doi:10.1021/acsnano.5c04980)
Supplement: Supplementary file 1 — nn5c04980_si_001.pdf [file nn5c04980_si_001.pdf]

Supplementary Information for

A Measurement Platform for Spin-Filtered Electron Currents that Probes the CISS Mechanism and Provides Directional Measurements for a Helical Conductive Fiber

Joseph A. Albro†, Noah T. Garrett†, Keerthana Govindaraj, Brian P. Bloom, Nathaniel L. Rosi, David H. Waldeck*

†These authors contributed equally to this work.

Figure S1 shows the molecular structure of chiral PANI (top), absorbance (bottom, left) and corresponding circular dichroism (bottom, right) of S-PANI (blue) and R-PANI fibers (red) measured in a 1:3 mixture of THF and $CHCl_3$. The transitions at 350, 450, and 700 nm in the absorbance spectra are consistent with that found in other works[1,2] and are attributed to π-π transitions between the benzenoid rings, polaron absorption, and excitation of electrons between benzenoid and quinoid rings, respectively. The mirror image Cotton effects at the electronic transitions of the polymer show opposite signs for S- and R-PANI and indicate chiral imprinting onto the electronic states of the fiber.

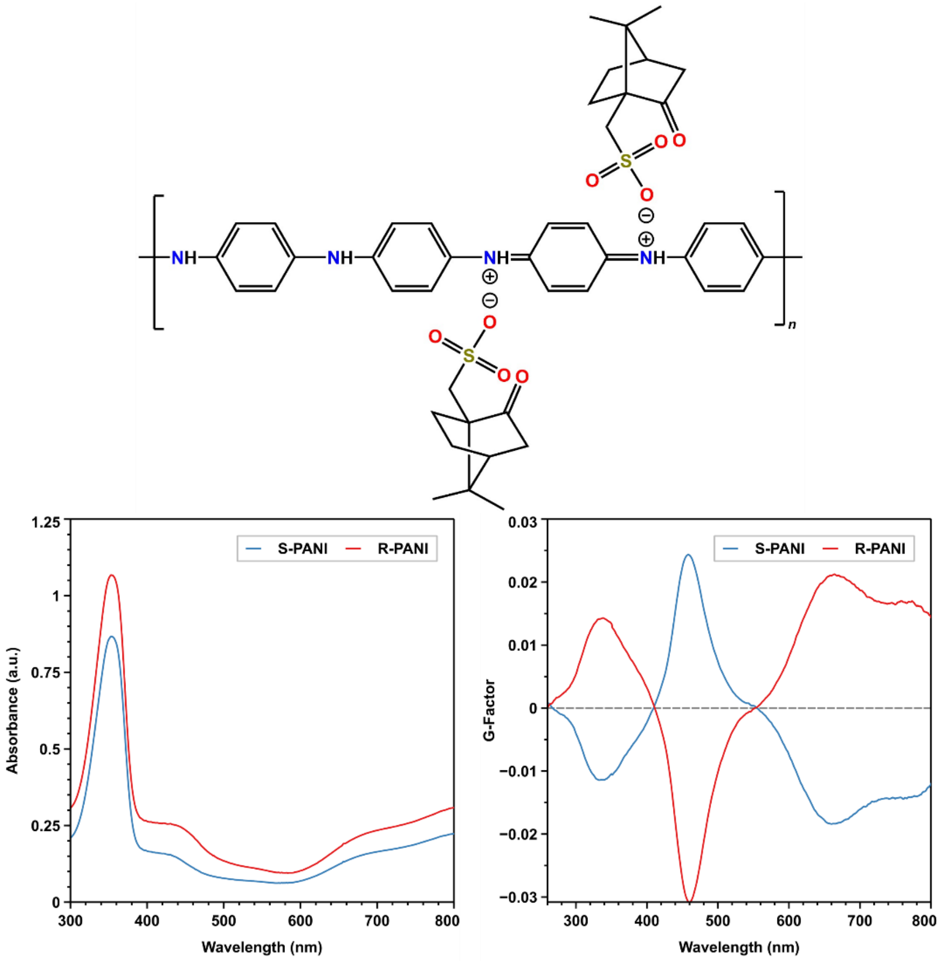

Figure S1. Molecular structure (top), absorbance (bottom, left) and circular dichroism (bottom, right) of R-(red) and S-PANI fibers (blue).

Figure S2 shows a vector plot of the magnetic field, B, of the magnet assembly simulated using Finite Element Method Magnetics[3] and demonstrates that the magnetic field is oriented in the Z direction, in the region where the measurement platform is placed. The inset provides an expanded view of the sample area. Arrows point in the direction of the magnetic field, and the arrow length is proportional to the magnetic field strength. The spacing between the magnets is approximately 12.7 mm. Simulation of the magnetic field strength along the sample provides a minimum field strength of 130 mT (at the sample height and position between the two magnets), in good agreement with the measurements that were made with a gaussmeter.

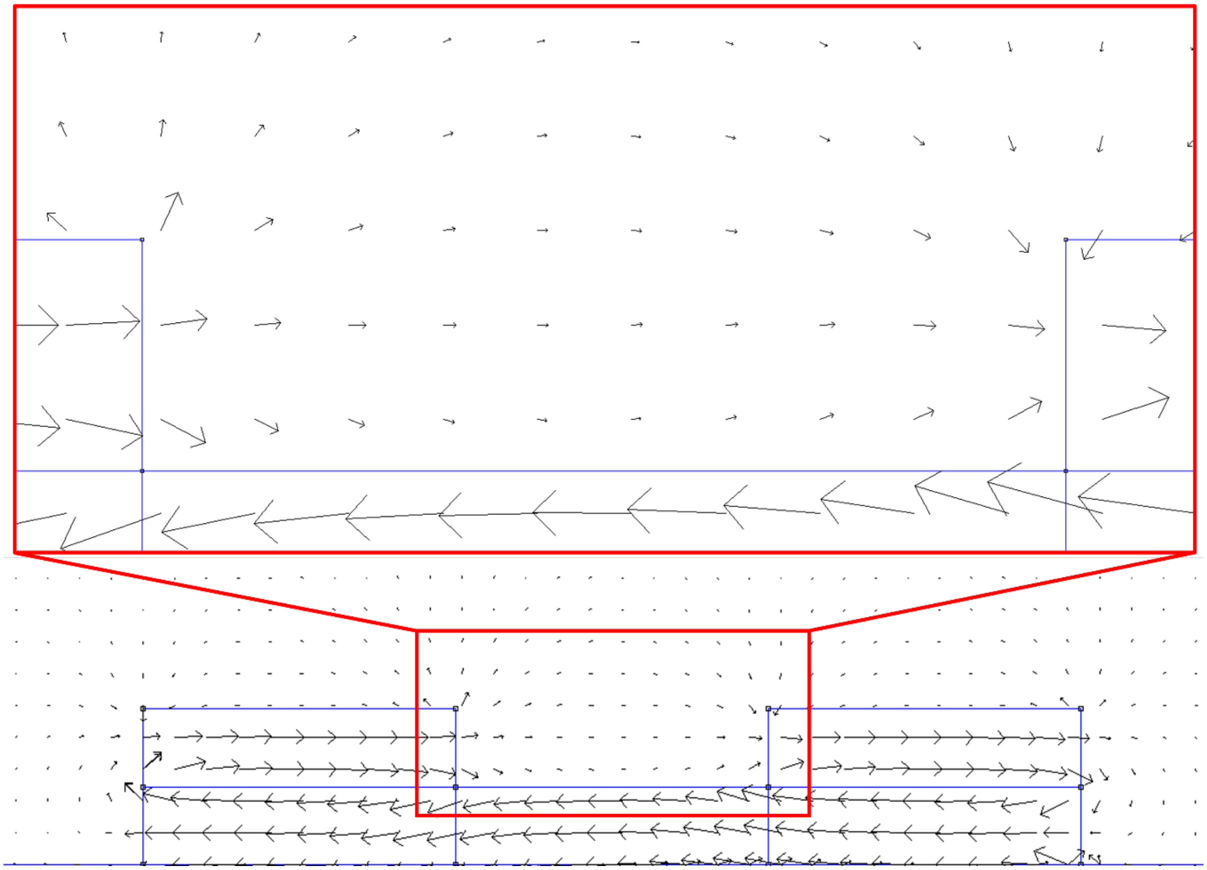

Figure S2. Magnetic field vector lines from simulation of the magnet assembly using Finite Element Method Magnetics.

Figure S3 shows corresponding SEM images of R-PANI (left) and S-PANI fibers (right). The orange highlighted region in the image illustrates the opposite helicity for the different enantiomers.

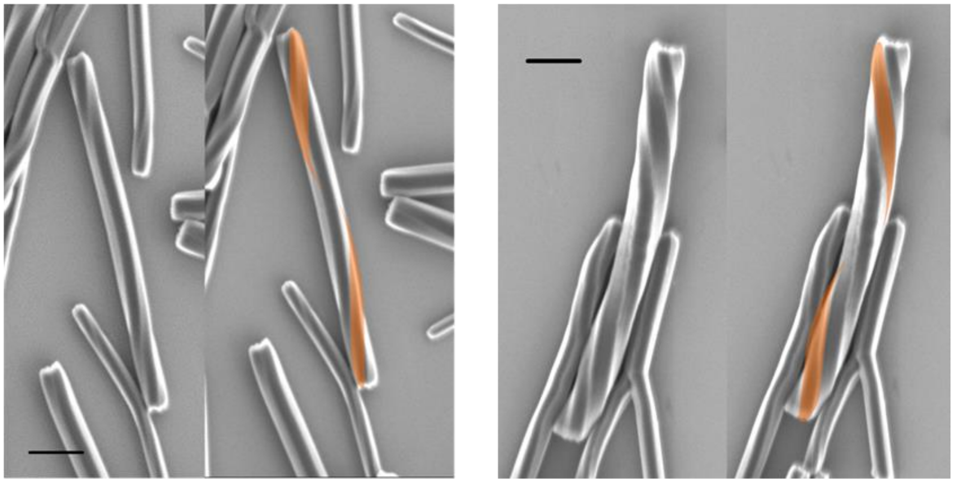

Figure S3. SEM images of R-PANI (left) and S-PANI (right). The chiral superstructure is highlighted in orange for both chirality fibers. The scale bar corresponds to 2 μm.

Figure S4 shows spin polarization measurements made on an R-PANI fiber with a B(+Z) applied magnetic field. The blue trace corresponds to measurements made to the left of the electrode and the red trace corresponds to measurements made to the right of the electrode. An opposite preference to that of the S-PANI fiber shown in Figure 4 is observed under the same applied magnetic field. Note that, the difference in conductivity between the S-PANI fiber in Figure 4 compared to that of the R-PANI fiber shown in Figure S4 is responsible for the different magnitude of current response. This feature is not unique to the handedness of the fiber, rather it arises from variability in the observed fiber-to-fiber conductance.

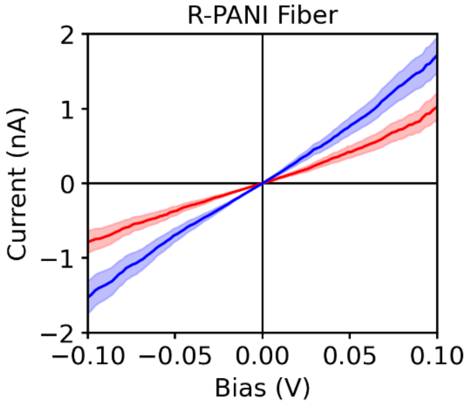

Figure S4. Spin polarization measurements on an R-PANI fiber measured under B(+Z) magnetic field direction. With measurements made on the left (blue) and right (red) of the fiber. The shaded region corresponds to the 95% confidence interval to the data.

Figure S5 shows additional longitudinal and transverse spin polarization measurements of an S-PANI fiber. The longitudinal spin polarization remains approximately the same when the magnetic field is reversed (B(+Z): 67 ± 5.8%, B(-Z): 69 ± 8.5%), and it is significantly higher than the transverse spin polarization measured on the same fiber (27 ± 5.4%). Locations used to measure the spin polarization on the fiber to the left are shown in blue, to the right in red, and locations for B(+X) are shown in brown while

the locations used for B(-X) are shown in green. Magnetoresistances for the red and blue side of the fiber are 72 ± 7.9% and -63 ± 6.2% respectively.

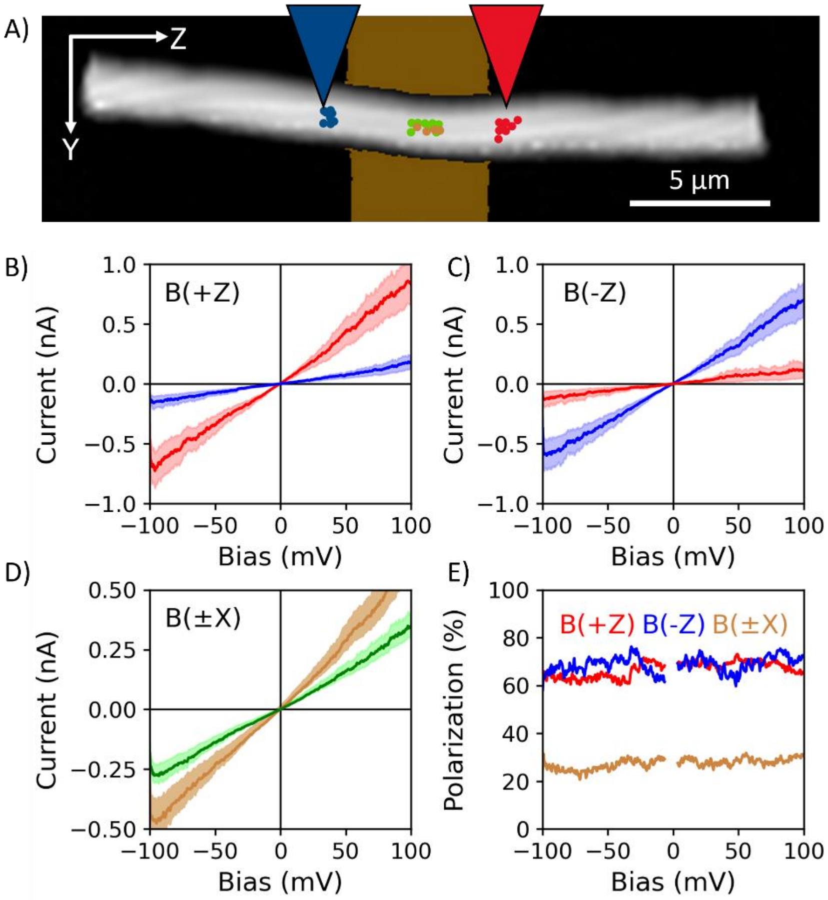

Figure S5. Measurement of an S-PANI fiber. A) AFM topography of the measured S-PANI fiber. Locations of longitudinal *i-V* measurements are shown by red and blue dots. The locations for the transverse *i-V* measurements are shown by green and orange dots and are approximately in the middle of the FM electrode. Blue and red colored AFM probes depict the side of the electrode corresponding to results shown in panels B) &C). An average of 120 *i-V* curves from 4 different locations for each side of the fiber are reported for the magnetic field oriented in the +Z direction, B(+Z), (Panel B) and the -Z direction, B(-Z), (Panel C). Panel D) reports an average of 60 *i-V* curves with magnetization out-of-plane along the X direction; B(+X) and B(-X) are represented by brown and green curves respectively. The shaded region in all of the curves represents 95% confidence intervals to the average data. E) Spin polarization as a function of sample bias.

Figure S6 shows additional longitudinal spin polarization measurements of an R-PANI fiber under B(+Z), B(+X), and B(-X) oriented magnetic fields. The spin polarization under B(+Z) is -46 ± 3.6%, while the polarization measured at positions of approximately 1 micron from the FM electrode edge for B(+X) is

-2.7 ± 3.4%, and B(-X) is 2.7 ± 4.5%. Locations used to measure the spin polarization on the fiber to the left are shown in blue and to the right in red. Calculated magnetoresistance for the red and blue side of the fibers is 14 ± 4.0% and 19 ± 5.3%, respectively.

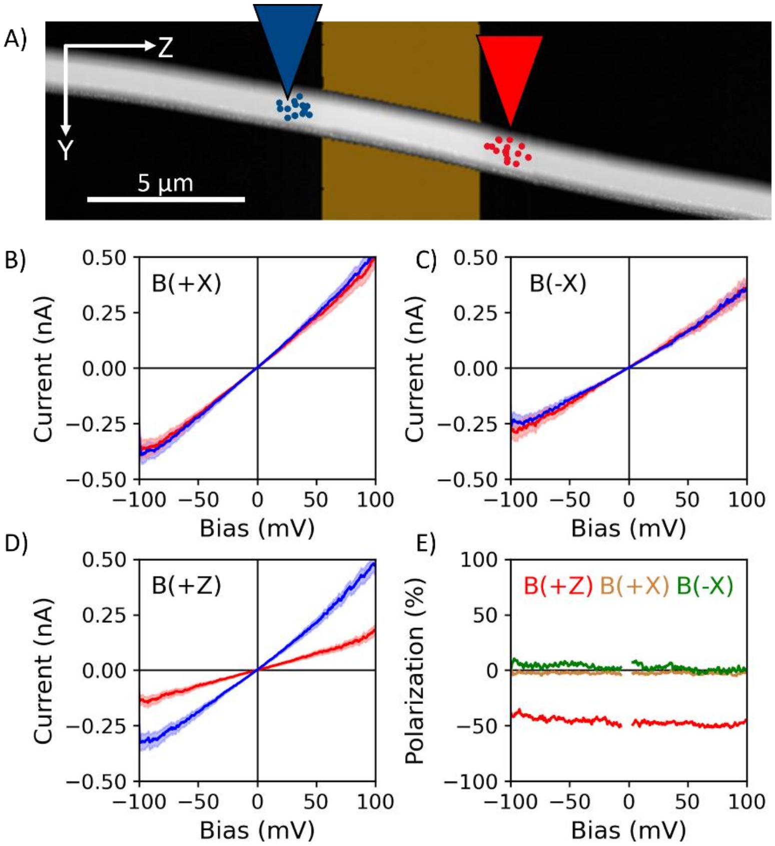

Figure S6. Longitudinal measurements of an R-PANI fiber under multiple field directions. A) AFM topography of the measured R-PANI fiber. Locations of longitudinal *i-V* measurements are shown by red and blue dots. Blue and red colored AFM probes depict the side of the electrode corresponding to results shown in Panels B), C), and D). An average of 120 *i-V* curves from 4 different locations for each side of the fiber are reported for the magnetic field oriented in the +X direction, B(+X), (Panel B), the -X direction, B(-X), (Panel C), and the magnetic field oriented in the +Z direction B(+Z), (Panel D). The shaded region in all of the curves represents 95% confidence intervals to the average data. Panel E) plots the spin polarization as a function of sample bias.

1 W. Zou, Y. Yan, J. Fang, Y. Yang, J. Liang, K. Deng, J. Yao, Z. Wei *J. Am. Chem. Soc.* **2014**, *136*, 578-581.

2 B. Kavitha, K. Prabakar, K. Siva Kumar, D. Srinivasu, Ch. Srinivas, V. K. Aswal, V. Siriguri, N.Narsimlu *IOSR J. Appl. Chem.* **2012**, *2* 16-19.

3 Meeker, D. Finite Element Method Magnetics, ver 4.2 (21 April 2019 Build), https://www.FEMM.info
